# Calculating incidence rates and prevalence proportions: not as simple as it seems

**DOI:** 10.1186/s12889-019-6820-3

**Published:** 2019-05-06

**Authors:** Inge Spronk, Joke C. Korevaar, René Poos, Rodrigo Davids, Henk Hilderink, François G. Schellevis, Robert A. Verheij, Mark M. J. Nielen

**Affiliations:** 1 Nivel, Netherlands Institute for Health Services Research, P.O. Box 1568, 3500BN, Utrecht, The Netherlands; 2 Department of Public Health, Erasmus MC, University Medical Center Rotterdam, Rotterdam, The Netherlands; 3 Centre for Health and Society, National Institute for Public Health and the Environment (RIVM), Bilthoven, The Netherlands; 4 Department of General Practice & Elderly Care Medicine/EMGO Institute for health and care research, VU University Medical Center, Amsterdam, The Netherlands

**Keywords:** Incidence rate, Prevalence proportion, General practice, Electronic health record

## Abstract

**Background:**

Incidence rates and prevalence proportions are commonly used to express the populations health status. Since there are several methods used to calculate these epidemiological measures, good comparison between studies and countries is difficult. This study investigates the impact of different operational definitions of numerators and denominators on incidence rates and prevalence proportions.

**Methods:**

Data from routine electronic health records of general practices contributing to NIVEL Primary Care Database was used. Incidence rates were calculated using different denominators (person-years at-risk, person-years and midterm population). Three different prevalence proportions were determined: 1 year period prevalence proportions, point-prevalence proportions and contact prevalence proportions.

**Results:**

One year period prevalence proportions were substantially higher than point-prevalence (58.3 - 206.6%) for long-lasting diseases, and one year period prevalence proportions were higher than contact prevalence proportions (26.2 - 79.7%). For incidence rates, the use of different denominators resulted in small differences between the different calculation methods (-1.3 - 14.8%). Using person-years at-risk or a midterm population resulted in higher rates compared to using person-years.

**Conclusions:**

All different operational definitions affect incidence rates and prevalence proportions to some extent. Therefore, it is important that the terminology and methodology is well described by sources reporting these epidemiological measures. When comparing incidence rates and prevalence proportions from different sources, it is important to be aware of the operational definitions applied and their impact.

## Background

Incidence rates and prevalence proportions of symptoms and diseases in the general population are important indicators of a population’s health status [1]. These epidemiological measures of disease frequency are the foundation to monitor diseases, formulate and evaluate healthcare policy and conduct scientific research [2]. The comparison of incidence rates and prevalence proportions between studies and countries, and determining factors explaining differences, results in increased knowledge on both prevention and aetiology of diseases [3]. However, fair comparisons between data sources are difficult to make due to differences induced by the use of different numerators and denominators.

From epidemiological handbooks, the definitions of incidence rates and prevalence proportions are not unambiguous. The incidence rate ‘represents the frequency of new occurrences of a medical disorders in the studied population at risk of the medical disorder arising in a given period of time’ and the prevalence proportion is ‘the part (percentage or proportion) of a defined population affected by a particular medical disorder at a given point in time, or over a specified period of time’ [4, 5]. Incidence is a rate of occurrence and thus related to a longitudinal design, whereas prevalence is the frequency of occurrence at a given point in time and connects to a cross-sectional sample [6]. However, further operationalisation of these definitions requires a number of decisions for both the denominator and numerator. In general, there is low level of consensus on which operationalisations are best and various methods are applied. Besides, in some circumstances the available information does not allow us to choose between different definitions [7]. Moreover, what was already highlighted by Elandt-Johnson in 1975 and which is still true nowadays, is that there is a lack of precision and ambiguity in terminology within the field of epidemiology [8]. Especially round the term ‘rate’ which is interchangeably used with the term proportion and sometimes with the term ratio [8, 9]. As a consequence, the comparability of incidence rates and prevalence proportions between different sources is challenging.

First, decisions are needed to establish the denominator. There are two main approaches used to define the patient population for the denominator, including the whole population in a year [10, 11], and the population at one specific point in time [12, 13]. For the calculation of incidence rates an at-risk population in a year is used as a third approach [14, 15]. Using person-years at risk is the correct method to calculate incidence rates according to the definition of incidence [4, 5, 16], however it is not always possible to adequately determine this population on the available information [7] and therefore also other denominators are used.

Second, for prevalence proportions, the definition of the prevalence proportion needs to be specified, which affects both the denominator and numerator. There are three definitions used: 1) a point-prevalence, the proportion of the population that has a disease at a specific point in time [17–19], 2) a 1 year period prevalence, the proportion of the population that has a disease at some time during a year [10, 20, 21] and 3) a contact prevalence, the proportion of the population with at least one encounter with a health care professional for a disease during a year [22–25].

These operational definitions will affect incidence rates and prevalence proportions but their impact is unknown. Therefore, the purpose of the current study is to investigate the impact of different operational definitions on incidence rates and prevalence proportions based on general practice data.

## Methods

### NIVEL primary care database

Data were derived from electronic health records (EHRs) of general practices contributing to NIVEL Primary Care Database (https://www.nivel.nl/en/nivel-primary-care-database). Data included consultations, morbidity, diagnostic tests, and drug prescriptions of all patients enlisted in these practices. Diagnoses were recorded and classified by general practitioners (GPs) according to the International Classification of Primary Care 1 (ICPC-1) [26]. Data from 2010 to 2012 including 408 general practices (reference date of extraction of the database: October 20, 2014) were used to calculate incidence rates and prevalence proportions for 2012. To ensure completeness and good quality of data, only data from practices meeting quality criteria were used [27].

### Denominator

Dutch inhabitants are obligatory linked to a general practice, including those persons who do not visit their associated GP. Therefore, the size, and age and gender distribution of the population can be determined from patient lists and the listed practice population represents the general population [2, 28].

### Numerator

The numerator of incidence rates and prevalence proportions represents the number of persons with a particular symptom or disease. For determining the number of incident and prevalent cases, GP recorded diagnostic information was used. In their EHRs, GPs can link diagnostic information to encounters or so-called episodes of care, defined as the period between the first and last encounter for a certain health problem. However, for calculating incidence rates and prevalence proportions, episode of illness, which ‘extends from the onset of symptoms to their complete resolution’, are needed [29]. With data from NIVEL Primary Care Database, an algorithm was developed to construct episodes of illness based on recorded diagnoses of encounters and episodes of care [27]. The input for the algorithm consisted of raw data from EHRs over the period 2010–2012, including encounters recorded in episodes of care, single diagnosis-coded encounters and date of diagnosis for all chronic diseases that started before January 1st 2010.

The first step of the development of the algorithm, was categorising all ICPC-1 codes in non-chronic (reversible) and chronic (non-reversible) diseases by a group of experts including researchers, epidemiologists, GPs and medical informaticians. For the analyses in this paper we only used the episodes of illness of 109 chronic diseases and 155 long-lasting non-chronic diseases. To estimate the number of incident and prevalent chronic cases in 2012, we used all encounters in the period 2010–2012 and the date of diagnosis that started before January 1st 2010 of recorded episodes of care. The start date of the episode is either the start date of the episode of care or the first encounter for this health problem in the period 2010–2012. For chronic diseases, no end date of the episode of illness is defined, since chronic diseases are considered irreversible. For the long-lasting non-chronic diseases, we used all recorded encounters and episodes of care in the period 2010–2012 to estimate incident and prevalent cases in 2012. To make a distinction between two consecutive episodes of illness for the same non-chronic disease, a minimum contact-free interval, i.e. a period in which it is likely that a patient does not visit the GP again if a disease is over, of 52 weeks was defined, depending on the assumed length of the disease episode. After this period of time, a new episode of illness may occur. The end date of the episode of illness was estimated as half of the contact-free interval (26 weeks) after the last encounter, since the patient is recovered between the date of the last encounter and a maximum of 52 weeks.

### Incidence rates and prevalence proportions

EHRs provide information about the number of quarters patients were registered in a general practice in a year. The number of quarters registered is used to calculate the denominators. Most patients were registered for a whole year (90%), but due to moving, changing GP, death or birth, patients could be registered less than four quarters. Therefore, the term ‘person-year’ was used, which was defined as the number of quarters of the year that a patient was registered in a general practice.

Incidence rates were calculated as the sum of all new episodes of illness of a certain disease in 2012 divided by the size of the population. The size of the population was defined in three ways: 1) the total population in a year in person-years, 2) the midterm population, defined as the size of the population on July 1st, 3) the number of patient years of the population at-risk in a year (Table 1). The at-risk period is the period that a patient was not recorded having a specific disease, i.e. the time that the patient is at-risk for getting that disease. Prevalent cases are thus not included in the population at-risk. When the population in a year or the population at one point time is used, the denominator is the same for each diagnose, whereas the denominator was calculated for each diagnose separately if the at-risk population was used.

**Table 1:** Definitions of Numerators and Denominators

	Numerator	Denominator
Incidence rate		
Incidence rate: person-years	Sum of all new episodes of illness in 2012	Person-years
Incidence rate: person-years at-risk	Sum of all new episodes of illness in 2012	Person-years at-risk
Incidence rate: midterm population	Sum of all new episodes of illness in 2012	Midterm population
Prevalence proportion		
Point-prevalence proportion	Episodes on December 31th	Population on December 31th
1 year period prevalence proportion	Episodes in 2012	Person-years
Contact prevalence proportion	Number of persons with ≥1 contact in 2012	Person-years

Year and point-prevalence proportions were calculated as the sum of all patients with a particular episode of illness divided by the population (Table 1). We used person-years as the denominator for 1 year period prevalence proportions and the size of the population on December 31th 2012 was used for point-prevalence proportions. The numerator for 1 year period prevalence proportions included all patients with an episode of illness in 2012, for point-prevalence proportions the numerator was the sum of patients with an on-going episode of illness on December 31th 2012. We also calculated contact prevalence proportions. These were calculated as the sum of all patients with at least one encounter with a general practitioner for a particular disease in 2012 divided by person-years. Incidence rates and prevalence proportions were calculated per 1000 persons or per 1000 person-years, whichever was appropriate. The ten highest incident and prevalent cases were tabulated. All calculations were performed using Stata 13.0.

## Results

### Population characteristics

After exclusion of practices that did not satisfy the quality criteria, the study population consisted of 312 general practices (76%) (Table 2) which were geographically evenly distributed over the Netherlands and formed a representative sample of Dutch general practices according to urbanization level of the practice location. The total number of registered patients was 1,223,818 representing 1,145,726 person-years. The mean age of the population was 40.0 ± 22.8 years and consisted of slightly more females (50.7%) than males. Population characteristics were representative for the Dutch population with respect to age and sex [30]. The population on July 1st, 2012 (the midterm population) consisted of 1,130,532 patients and on December 31th of 1,105,536 patients.

**Table 2:** Characteristics of the Study Population

	Number of cases (n)	Percentage (%)
Population characteristics		
Patients	1,223,818	
Person-years	1,145,726	
Gender		
Male	603,179	49.3
Female	620,639	50.7
Age^a^ (year)		
0–4	63,969	5.2
5–17	190,197	15.5
18–44	432,438	35.3
45–64	338,904	27.7
65–74	111,286	9.1
75–84	62,395	5.1
≥ 85	24,629	2.0
General practice characteristics		
Number	312	
Patients (mean ± SD)	3923 ± 2449	
Person-years (mean ± SD)	3672 ± 2289	
Mode of practice^b^		
Solo	133	42.6
Duo	79	25.3
Group	76	24.4
Unknown	24	7.7
Degree of urbanization^c^		
Extremely urbanised	65	20.8
Strongly urbanised	71	22.8
Moderately urbanised	57	18.3
Hardly urbanised	60	19.2
Not urbanised	48	15.4
Unknown	11	3.5

^a^The age of patients on the last day of the year was used for the total population

^b^In a solo practice, one GP is working. In a duo practice two GPs are employed and in a group practice three or more GPs are engaged with the practice

^c^Extremely urbanized comprised of ≥2500 addresses/ km^2^; strongly urbanized of ≥1500–2500 addresses/ km^2^; moderately urbanised of ≥1000–1500 addresses/ km^2^; hardly urbanised of ≥500–1000 addresses/ km^2^; not urbanised of < 500 addresses/km^2^

### Incidence rates

Incidence rates of the ten highest incident diagnoses were calculated based on three different defined populations (Table 3). The use of person-years at-risk as denominator resulted in slightly higher rates compared to the use of person-years (0.9 - 14.8%). The differences were higher in chronic diagnoses than in long-lasting diagnoses.

**Table 3:** Incidence rates based on different denominators

Incidence rate /1,000	Numerator:	All new episodes in 2012	All new episodes in 2012	All new episodes in 2012	Difference person-years at-risk – person-years	Difference person-years at-risk – population on 1 July	Difference person-years – population on 1 July
	Denominator:	Person-years at–risk^a^	Person-years^b^	Population on 1 July^c^			
Long-lasting diagnosis^*^	ICPC	Mean	Mean	Mean	Mean (%)	Mean (%)	Mean (%)
Contact dermatitis/ allergic eczema	S88	34.78	33.83	34.28	0.95 (2.8%)	0.50 (1.4%)	-0.45 (-1.3%)
Hayfever/allergic rhinitis	R97	24.14	23.43	23.74	0.70 (3.0%)	0.39 (1.7%)	-0.31 (-1.3%)
Constipation	D12	20.75	20.36	20.63	0.39 (1.9%)	0.11 (0.6%)	-0.27 (-1.3%)
Naevus/mole	S82	16.11	15.96	16.17	0.15 (0.9%)	-0.06 (-0.8%)	-0.21 (-1.3%)
Lumbar disc lesion, back pain with radiating pain	L86	15.13	14.92	15.12	0.21 (1.4%)	0.01 (0.1%)	-0.20 (-1.3%)
Vitamin deficiency/other nutritional disorder	T91	12.90	12.74	12.91	0.16 (1.2%)	-0.01 (-0.1%)	-0.17 (-1.3%)
Shoulder syndrome	L92	11.97	11.86	12.01	0.11 (0.9%)	-0.05 (-0.4%)	-0.16 (-1.3%)
Depressive disorder	P76	10.70	10.51	10.65	0.20 (1.9%)	0.06 (0.5%)	-0.14 (-1.3%)
Allergy/allergic reaction	A12	10.28	10.19	10.33	0.09 (0.9%)	-0.05 (-0.4%)	-0.14 (-1.3%)
Cataract	F92	9.83	9.76	9.89	0.07 (0.7%)	-0.06 (-0.6%)	-0.13 (-1.3%)
Chronic diagnosis^*^	ICPC						
Uncomplicated hypertension	K86	12.59	10.96	11.11	1.63 (14.8%)	1.48 (13.3%)	-0.15 (-1.3%)
Atopic dermatitis/other eczema	S87	11.75	10.91	11.06	0.84 (7.7%)	0.69 (6.3%)	-0.15 (-1.3%)
Lipid metabolism disorder	T93	8.50	7.97	8.08	0.52 (6.5%)	0.42 (5.1%)	-0.11 (-1.3%)
Asthma	R96	8.14	7.49	7.59	0.65 (8.7%)	0.55 (7.2%)	-0.10 (-1.3%)
Refractive errors	F91	4.99	4.88	4.95	0.10 (2.1%)	0.04 (0.1%)	-0.06 (-1.3%)
Diabetes mellitus	T90	4.78	4.50	4.56	0.28 (6.3%)	0.22 (4.9%)	-0.06 (-1.3%)
Acquired deformity of limbs	L98	4.39	4.27	4.33	0.12 (2.8%)	0.06 (1.5%)	-0.06 (-1.3%)
Atherosclerosis (excl. K76,K90)	K91	4.18	4.15	4.20	0.04 (0.9%)	-0.02 (-0.5%)	-0.06 (-1.3%)
Malignant neoplasm of skin	S77	3.82	3.74	3.78	0.09 (2.3%)	0.04 (1.0%)	-0.05 (-1.3%)
Osteoarthritis knee	L90	3.72	3.63	3.68	0.09 (2.6%)	0.04 (1.2%)	-0.05 (-1.3%)

^*^The ten highest incident long-lasting and chronic diagnoses are displayed

^a^Number is per 1,000 person-years at-risk, ^b^Number is per 1,000 person-years, ^c^Number is per 1,000 persons

Comparing the use of person-years at-risk with the midterm population, incidence rates are for some diseases higher when the population at-risk is used. For other diseases, rates are higher when the midterm population was used. Differences ranged from − 0.8 to 13.3%.

When comparing the use of person-years with the midterm population, higher rates were found when the midterm population (difference − 1.3%). Absolute differences were low; ranging from − 0.05/1000 per year in chronic diseases to − 0.45/1000 per year in long-lasting diseases. For all three comparisons, differences were larger in high frequent diagnoses and smaller in low frequent diagnoses (results not shown).

### Prevalence proportions

Comparing 1 year period prevalence proportions with point-prevalence proportions on December 31th, substantially higher proportions were found for 1 year period prevalence proportions of long-lasting diseases (differences: 58.3–206.6%) (Table 4). On the contrary, point-prevalence proportions resulted in slightly higher rates (difference 3.5%) in chronic diagnoses. Absolute differences ranged from − 5.04/1000 per year in chronic diseases to 33.72/1000 per year in long-lasting diseases.

**Table 4:** Comparison of prevalence proportions calculated with different methods

Prevalence proportion /1.000		1 year period prevalence^a^	Point prevalence^b^	Contact prevalence^a^			
	Numerator:	Existing episodes in 2012	Existing episodes on 31 Dec 2012	Number of persons with ≥1 contact in 2012	Difference 1 year period prevalence point prevalence	Difference 1 year period prevalence contact prevalence	Difference point prevalence contact prevalence
	Denominator:	Person-years	Population on 31 Dec 2012	Person-years			
Long-lasting diagnosis^c^	ICPC	Mean	Mean	Mean	Mean (%)	Mean (%)	Mean (%)
Contact dermatitis/ allergic eczema	S88	58.41	24.68	41.31	33.72 (136.6%)	17.09 (41.4%)	−16.63 (−40.3%)
Hayfever/allergic rhinitis	R97	47.96	21.77	35.28	26.19 (120.3%)	12.68 (35.9%)	−13.51 (−38.3%)
Constipation	D12	37.94	18.28	25.57	19.66 (107.5%)	12.37 (48.4%)	−7.29 (−28.5%)
Lumbar disc lesion. Back pain with radiating pain	L86	28.31	13.63	20.50	14.68 (107.7%)	7.81 (38.1%)	−6.87 (−33.5%)
Depressive disorder	P76	28.25	17.84	21.62	10.41 (58.3%)	6.62 (30.6%)	−3.78 (−17.5%)
Naevus/mole	S82	24.73	9.51	17.04	15.22 (160.1%)	7.69 (45.1%)	−7.53 (−44.2%)
Vitamin deficiency/other nutritional disorder	T91	22.33	12.55	17.70	9.78 (77.9%)	4.64 (26.2%)	−5.14 (−39.1%)
Shoulder syndrome	L92	20.81	8.79	14.62	12.02 (136.7%)	6.19 (42.4%)	−5.83 (−39.9%)
Tobacco abuse	P17	18.52	6.04	10.30	12.48 (206.6%)	8.21 (79.7%)	−4.26 (−41.4%)
Allergy/allergic reaction	A12	18.47	8.13	12.31	12.31 (127.2%)	6.16 (50.0%)	−4.18 (−34.0%)
Chronic diagnosis^c^	ICPC						
Uncomplicated hypertension	K86	139.92	144.96	94.78	−5.04 (−3.5%)	45.14 (47.6%)	50.18 (52.9%)
Asthma	R96	87.46	90.60	39.23	−3.15 (− 3.5%)	48.23 (122.9%)	51.37 (130.9%)
Atopic dermatitis/other eczema	S87	79.82	82.68	23.77	−2.86 (−3.5%)	56.05 (235.8%)	58.91 (247.8%)
Lipid metabolism disorder	T93	67.81	70.25	30.41	−2.44 (−3.5%)	37.40 (123.0%)	39.84 (131.0%)
Diabetes mellitus	T90	64.12	66.43	55.70	−2.32 (−3.5%)	8.41 (15.1%)	10.73 (19.3%)
Acquired deformity of limbs	L98	31.07	32.18	5.99	−1.11 (−3.5%)	25.07 (418.4%)	26.19 (436.9%)
Emphysema/chronic obstructive pulmonary disease	R95	29.72	30.79	20.47	−1.07 (−3.5%)	9.25 (45.2%)	10.32 (50.4%)
Osteoarthritis knee	L90	27.90	28.91	9.64	−1.00 (−3.5%)	18.26 (189.4%)	19.26 (199.8%)
Malignant neoplasm of skin	S77	25.71	26.64	8.98	−0.92 (−3.5%)	16.73 (186.3%)	17.66 (196.6%)
Angina pectoris	K74	25.39	26.30	12.31	−0.91 (−3.5%)	13.08 (106.3%)	14.00 (113.7%)

^a^Number is per 1000 person-years, ^b^Number is per 1000 persons

^c^The ten highest prevalent long-lasting and chronic diagnosis are displayed

When 1 year period prevalence proportions were compared to contact prevalence proportions, largest differences were found for prevalence proportions of chronic diseases. These differed from 15.1% to 418.4% for high frequent chronic diseases. Also differences in long-lasting diseases were relevant. 1 year period prevalence proportions were 26.2–79.7% higher. Absolute differences ranged from 4.64/1000 per year in long-lasting diseases to 56.05/1000 per year in chronic diseases.

Finally, point-prevalence proportions were compared to contact prevalence proportions. Contact prevalence proportions were higher for long-lasting diseases (17.5–44.2%), whereas point-prevalence proportions were higher for chronic diseases (19.3–436.9%). Absolute differences ranged from -16.63/1000 per year in long-lasting diseases to 58.91/1000 per year in chronic diseases. For all three comparisons, differences were larger in low frequent diagnoses and smaller in high frequent diagnoses (results not shown).

## Discussion

This study investigated to what extent different operational definitions of the numerator and denominator influence incidence rates and prevalence proportions. Different definitions to define the population denominator have a small effect on incidence rates. However, the use of an 1 year period prevalence proportion instead of a point-prevalence or contact prevalence results in large differences. Authors should therefore thoroughly report how they have calculated their presented epidemiological numbers. Besides, to ensure comparability of point-prevalence proportions from different studies, the time point used in the study should be reported.

Valid incidence rates and prevalence proportions are important as they are the foundation to monitor diseases and they are used to formulate and reflect on healthcare policy [2]. Comparison of these epidemiological measures between different sources, like between different countries, is important as well as investigation on factors explaining differences lead to increased knowledge on both aetiology and prevention of diseases [3]. Operational definitions of the numerator and denominator to calculate incidence rates and prevalence proportions are of influence to the actual rates and proportions and therefore it is important to be aware of these influences in order to make fair comparisons.

Theoretically, the use of person-years results in a more reliable denominator for incidence rates than the midterm population. Incidence rates include a time component which is not incorporated in a fixed population, and therefore, a population at one point in time is not appropriate. Furthermore, person-years take into account incomplete follow-up and results thereby in a more precise denominator. However, the number of person-years at-risk is the only correct reliable denominator as it corresponds best to the definition of incidence rates [4, 5, 16]. It is the only denominator that takes into account the time that a person suffers from a specific disease. This time should not be included in the denominator as the person is not at-risk of developing that disease during that time [4, 5, 16]. In fact, when using another definition of the denominator than person-years at-risk, it should be called an incidence proportion instead of an incidence rate [8]. However, all three used denominators in this study are used in general practice-based epidemiological research. In studies based on data from general practices in countries without a patient list, a population at one point in time is often used, as it is hard to define a reliable denominator in these countries [7]. Studies from general practices in countries with a patient list are not consistent in defining the denominator and use either person-years [21, 31–33] or person-years at-risk [34–36]. Based on the results of this study, it can be concluded that using different definitions of the population (i.e. different denominators) results in relevant differences in incident rates, especially in frequent and in highly frequent diseases.

In general practice-based epidemiological research, 1 year period prevalence proportions, point-prevalence proportions as well as contact prevalence proportions are reported. Our results show clear differences between these three types of prevalence proportions. The most striking impact for long-lasting diagnoses was the decision for 1 year period prevalence proportions instead of point-prevalence proportions; 1 year period prevalence proportions were more than twice as high. Among prevalence proportions of chronic diagnoses, the largest differences were seen when a 1 year period prevalence proportion was calculated instead of a contact prevalence proportion.

One year period prevalence proportions are most often used in general practice research. The major differences between 1 year period prevalence proportions and point-prevalence proportions on December 31th are caused by the number of persons with an ending episode in the course of a year for long-lasting diseases. When calculating an 1 year period prevalence proportion, all existing episodes in a year contribute to the numerator. Whereas in a point-prevalence the existing episodes on an indicated date are summed. The number of persons with an existing episode in a year is substantially higher than the number of persons with an existing episode on December 31th, explaining the large differences in prevalence proportions for long-lasting diseases. For chronic diseases, this does not apply as chronic diseases are non-reversible. The numerator only slightly differs through people that are deceased or moved. And as the number of people registered during the year in person-years are higher than the number of people registered on December 31th, point-prevalence proportions are slightly higher than 1 year period prevalence proportions for chronic diseases.

The substantially higher 1 year period prevalence proportions compared to contact prevalence proportions are caused by the numerator, since for both prevalence proportions the denominator is the number of person-years. For 1 year period prevalence proportions, existing and new episodes are summed in the numerator, whereas for contact prevalence proportions, the number of persons with a contact for a specific disease are summed. The difference is caused by episodes of illness without an encounter in the forthcoming year. Differences were in particular higher for chronic diseases. This is caused by the fact that chronic diseases have a life-long history and people may not visit their GP for a while. People may not suffer that much to visit the GP in a particular year, or they are solely visiting secondary care for their chronic disease. This is how using contact prevalence proportions can introduce errors. Especially for chronic diseases, the contact prevalence proportion can largely differ from that of other prevalence proportions because the contact prevalence depends on the condition and on the amount of care a patient needs. Some conditions increase utilization of GP care while others do not. This is important to keep in mind when considering the use of contact prevalence proportions.

Next to the importance of differences in incidence rates and prevalence proportions calculation, also differences in the studied population (for example in age, sex, socio-economic class, ethnic background etc.) could result in large differences in presented incidence rates and prevalence proportions. Which also make comparisons across studies harder. Standardization of rates to age and sex will help to overcome this issue.

A strength of current study is that we were able to apply all different operational definitions of incidence rates and prevalence proportions on the same dataset. Therefore, other causes contributing to differences in rates and proportions, like differences between databases and between populations [37, 38], did not influence the epidemiological measures. A limitation is the focus on long-lasting and chronic diseases. Operational definitions for incidence rates could also been investigated for acute diagnoses, but as 1 year prevalence proportions and contact prevalence proportions are comparable due to the short minimum contact-free interval of acute diagnosis this comparison is less interesting. Besides, point-prevalence proportions are less interesting as well through the seasonal influences of acute diagnosis. Another limitation is the fact that the used general practice data is not 100% complete. Only data from practices meeting quality criteria were used in present study. This ensures good quality of data, but it does not guarantee completeness of data. We do not think that this limitation influenced our results as we studied differences between incidence rate and prevalence proportions; we did not focus on the incidence rates or prevalence proportions of specific diagnosis. Another limitation is the possible bias introduced by using quarters of a year to define the denominator. However, our patient population can only be defined by health care claims by the GP. For each patient, a GP claims a certain amount of money each quarter. We do not think this has a large impact on our findings, as around 90% of the population is registered the complete year in a practice.

## Conclusion

Operational definitions of denominators and numerators to calculate incidence rates and prevalence proportions influence these epidemiological measures to some extent and thereby affect the comparability of studies. Using different denominators accounts for only slight differences in incidence rates. In contrast, the decision for the type of prevalence has high impact on prevalence proportions. It is therefore important that both the terminology and methodology is well described by sources reporting these epidemiological measures. When comparing incidence rates and prevalence proportions from different sources, it is very important to be aware of the operational definitions applied and their impact.

## Abbreviations

EHRs: Electronic health records
GP: General practitioner
ICPC-1: International Classification of Primary Care 1
